# Evaluation of Truck Cab Decontamination Procedures following Inoculation with Porcine Epidemic Diarrhea Virus and Porcine Reproductive and Respiratory Syndrome Virus

**DOI:** 10.3390/ani14020280

**Published:** 2024-01-16

**Authors:** Grace E. Houston, Cassandra K. Jones, Caitlin Evans, Haley K. Otott, Charles R. Stark, Jianfa Bai, Elizabeth G. Poulsen Porter, Marcelo N. de Almeida, Jianqiang Zhang, Phillip C. Gauger, Allison K. Blomme, Jason C. Woodworth, Chad B. Paulk, Jordan T. Gebhardt

**Affiliations:** 1 Department of Diagnostic Medicine/Pathobiology, College of Veterinary Medicine, Kansas State University, Manhattan, KS 66506-0201, USA; 2 Department of Animal Sciences and Industry, College of Agriculture, Kansas State University, Manhattan, KS 66506-0201, USA; jonesc@ksu.edu (C.K.J.); jwoodworth@ksu.edu (J.C.W.); 3 Department of Grain Science and Industry, College of Agriculture, Kansas State University, Manhattan, KS 66506-0201, USA; ceevans@ksu.edu (C.E.); haley27@ksu.edu (H.K.O.); ablomme@ksu.edu (A.K.B.); cpaulk@ksu.edu (C.B.P.); 4 Department of Veterinary Diagnostic and Production Animal Medicine, College of Veterinary Medicine, Iowa State University, Ames, IA 50011-1134, USA; malmeida@iastate.edu (M.N.d.A.); jqzhang@iastate.edu (J.Z.); pcgauger@iastate.edu (P.C.G.)

**Keywords:** disinfectants, porcine epidemic diarrhea virus, porcine reproductive and respiratory syndrome virus, truck cabs, swine

## Abstract

**Simple Summary:**

It is widely understood that transportation vehicles can serve as potential vectors of pathogen transmission within swine production systems for pathogens of concern such as porcine epidemic diarrhea virus (PEDV) and porcine reproductive and respiratory syndrome virus (PRRSV). The objective of this experiment was to evaluate several decontamination methods for the mitigation of PEDV and PRRSV within truck cabs. A total of three full-sized truck cabs were modified for use in a BSL-2 research facility with multiple surface types including fabric, rubber, and plastic. Surfaces were inoculated with either PEDV alone, PRRSV alone, PEDV + an organic matter mixture of feces and dirt, or PRRSV + organic mixture. Practical decontamination methods were then applied using a pump sprayer or Hurricane fogger system, or a commercially available gaseous chlorine dioxide system. Several differences were observed within the different combinations of disinfectants and surfaces, indicating that under the conditions of this study, the ability of different disinfectants to reduce the detection of PEDV and PRRSV genetic materials differed depending on the surface being evaluated. In general, most disinfectant applications were only able to reduce the quantity of detectable virus but not completely eliminate it from the surface.

**Abstract:**

This experiment aimed to evaluate commercially available disinfectants and their application methods against porcine epidemic diarrhea virus (PEDV) and porcine reproductive and respiratory syndrome virus (PRRSV) on truck cab surfaces. Plastic, fabric, and rubber surfaces inoculated with PEDV or PRRSV were placed in a full-scale truck cab and then treated with one of eight randomly assigned disinfectant treatments. After application, surfaces were environmentally sampled with cotton gauze and tested for PEDV and PRRSV using qPCR duplex analysis. There was a disinfectant × surface interaction (*p* < 0.0001), indicating a detectable amount of PEDV or PRRSV RNA was impacted by disinfectant treatment and surface material. For rubber surfaces, 10% bleach application had lower detectable amounts of RNA compared to all other treatments (*p* < 0.05) except Intervention via misting fumigation, which was intermediate. In both fabric and plastic surfaces, there was no evidence (*p* > 0.05) of a difference in detectable RNA between disinfectant treatments. For disinfectant treatments, fabric surfaces with no chemical treatment had less detectable viral RNA compared to the corresponding plastic and rubber (*p* < 0.05). Intervention applied via pump sprayer to fabric surfaces had less detectable viral RNA than plastic (*p* < 0.05). Furthermore, 10% bleach applied via pump sprayer to fabric and rubber surfaces had less detectable viral RNA than plastic (*p* < 0.05). Also, a 10 h downtime, with no chemical application or gaseous fumigation for 10 h, applied to fabric surfaces had less detectable viral RNA than other surfaces (*p* < 0.05). Sixteen treatments were evaluated via swine bioassay, but all samples failed to produce infectivity. In summary, commercially available disinfectants successfully reduced detectable viral RNA on surfaces but did not eliminate viral genetic material, highlighting the importance of bioexclusion of pathogens of interest.

## 1. Introduction

Biosecurity and maintaining healthy populations of animals are critical for modern swine production. Key viral pathogens affecting commercial swine production include porcine reproductive and respiratory syndrome virus (PRRSV) and porcine epidemic diarrhea virus (PEDV). While PRRSV has been present in North America for decades, PEDV was first detected in North America in 2013. Specific estimates of economic impact are challenging to summarize, and the estimated economic impact of PRRSV in North America was USD 664 million as per a 2013 publication by Holtkamp et al. [1]. Additional research has been conducted to estimate the economic impact at the farm level in the United States [2], China [3], and Germany [4], and additional information is being generated regarding the impact of PRRSV on wean-finish mortality in commercial settings [5]. While PRRSV is one of the costliest diseases affecting swine production in North America, the rapid distribution of PEDV in the United States beginning in 2013 resulted in substantial industry losses [6]. Following the initial introduction into the United States in 2013, the incidence of PEDV on sow farms has been lower compared to the initial epidemic [7]; however, it remains a challenging virus to control especially in growing and finishing pig populations due to gaps in biosecurity practices.

Both viruses are enveloped, positive-stranded RNA viruses [8,9]. The transmission of PRRSV occurs through exposure via the respiratory and/or oral routes as well as through vertical transmission in gestating females [10]. The transmission of PEDV occurs through fecal–oral transmission [9]. The control of these pathogens involves attention to several different potential vectors including animal movement, supply entry into farms, personnel movement, and other fomites such as transportation vehicles. Research suggests that transportation vehicles can play a role in the transmission of bacterial [11] and viral pathogens [12,13,14,15]. Cleaning and disinfecting metal surfaces has been shown to reduce detectable PRRSV and PEDV [16,17,18,19,20,21]. These methods are successful at reducing detectable viral RNA, and techniques including power washing, disinfecting, and heating semi-truck trailers responsible for live animal transportation are commonplace in the current United States swine industry as biosecurity measures. However, when considering the role of feed delivery in pathogen introduction, the likelihood of detecting PEDV or porcine deltacoronavirus RNA was highest within the truck cab of the feed delivery truck as opposed to the trailer [22].

To date, truck cabs have been an under-evaluated potential source of pathogen transmission, and very little is known regarding the best practices for their decontamination. A challenge associated with the cab of feed delivery trucks is that multiple surface types are present within a single space. When considering the efficacy of disinfectants and surface types, a previous study has found that surface type can influence the detection of viral RNA after disinfectant treatment [23]. However, most disinfection protocols are developed by extrapolations from laboratory settings or are otherwise lacking for applying laboratory bench data to real-world settings. Therefore, the objective of this study was to evaluate commercially available disinfectants and application methods on different surfaces present within truck cabs for efficacy in reducing detectable PEDV and PRRSV RNA and subsequent infectivity of surface environmental samples.

## 2. Materials and Methods

The surface inoculation and sample collection for this study were conducted at the Cargill Feed Science Research Center (FSRC) at the O.H. Kruse Feed Mill of Kansas State University (KSU) in Manhattan, KS, USA with approval from the KSU Institutional Biosafety Committee (Project Approval #1511). The inoculation of surfaces was performed within a biosafety cabinet (BSC) housed within the BSL-2 space of the FSRC. This study was set up in an 8 × 3 × 2 factorial with eight disinfectant methods, three different surfaces, and two viruses, with each combination of factors repeated three times.

### 2.1. Preparation of Inoculum

Viral inoculum was propagated and titrated at the South Dakota State University Animal Disease Research & Diagnostic Laboratory using common virological techniques. Briefly, stock PEDV was cultured using Vero-76 cells, and for PRRSV, MARC-145 cells were used. Following propagation and virus titration, viral inoculum was prepared by placing 25 mL of PEDV (USA/CO/2013 isolate with a titer of 1.33 × 10^6^ TCID_50_/mL; GenBank accession number: KF272920) and 25 mL of PRRSV (1-7-4 isolate with a titer of 1.33 × 10^6^ TCID_50_/mL) into separate containers. In each container, 225 mL of phosphate-buffered solution (PBS) was added to achieve a final concentration of 10^5^ TCID_50_/mL. Each virus was divided into five containers containing approximately 45 mL volume, sealed and stored at −80 °C for two weeks. When virus was needed for the study, the containers of each virus were defrosted in a lukewarm water bath (approximately 36 °C to 40 °C) and used immediately. Inoculum samples were taken during the study to evaluate Ct values over time. When evaluating the Ct values of pure virus stock, the PRRSV stock was always one to two Ct values higher (Ct values ranging from 23.3 to 24.0) than the virus stock of PEDV (Ct values ranging from 22.1 to 22.3).

### 2.2. Preparation of Surfaces and Disinfectant

Plastic (0.32 × 10.16 × 20.32 cm white high-density polyethylene panel; Menards, Eau Claire, WI, USA), rubber (Heavy-Duty 45.72 × 45.72 × 0.64 cm Rubber Gym Tiles; BCG, Boston, MA, USA), and fabric surfaces (upholstery fabric, no water resistance treatment; Joann’s Fabrics, Hudson, OH, USA) were cut into 10 cm × 10 cm squares for the creation of surface coupons. There were a total of 48 coupons for each surface type (48 plastic, 48 rubber, 48 fabric). Velcro strips were applied to the back of the surface coupons prior to inoculation, and surface coupons were placed into a transportation container (Promoze Food Storage Containers, Seattle, WA, USA), and they remained in these storage containers until ready for placement into the truck cabs. Three truck cabs of similar make and model were sourced from a local salvage yard (Beloit, KS, USA). The truck cabs were removed from their frame at the salvage yard, transported to the FSRC for the study, and placed on wheels for easy mobility. Truck cabs were visually and physically inspected for any holes or inadequate seals and sealed as necessary using either silicone sealant (DAP; Menards, Eau Claire, WI, USA) or super glue (Gorilla Glue; Gorilla Glue Co, Sharonville, OH, USA). At the start of the study, truck cabs were wheeled into the BSL-2 space, their wheels were locked, and they remained in the same location for the duration of the study. Each truck had eight disinfectant treatments applied in random order. Disinfectant treatments included the following:

1. No disinfectant.
2. 1:256 dilution of Synergize (Neogen Corp, Lexington, KY, USA) applied through misting fumigation (Hurricane Ultra II Portable Electric Fogger, Curtis Dyna-Fog Ltd., Westfield, IN, USA).
3. 1:256 dilution of Synergize applied through pump sprayer (Chapin Sure Spray 1 Gallon Tank Sprayer, Menards, Eau Claire, WI, USA).
4. 1:64 dilution of Intervention (Virox, Oakville, ON, USA) applied through misting fumigation.
5. 1:64 dilution of Intervention applied through pump sprayer.
6. 10% bleach (7.55% sodium hypochlorite germicidal bleach; Clorox, Oakland, CA, USA) solution applied through pump sprayer.
7. No chemical treatment, 10 h holding time.
8. Gaseous fumigation over 10 h with chlorine dioxide (ProKure G; ProKure Solutions, Phoenix, AZ, USA).

The designated concentration of wet disinfectants was prepared daily. Once prepared, solutions were poured into their respective application method tool: pump sprayer or misting fumigation. The water-based chlorine dioxide pouch was utilized following manufacturer-labeled instructions for the gaseous fumigation application.

### 2.3. Surface Inoculation and Disinfectant Application

**Figure 1:**
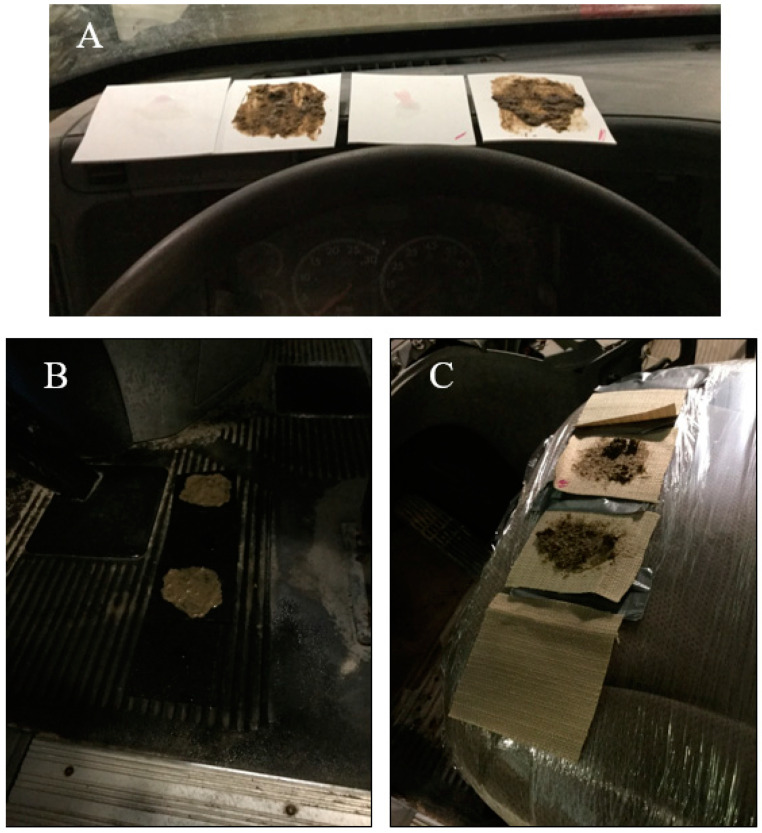
Surface inoculation for plastic (**A**), rubber (**B**), and fabric (**C**) coupons within full-sized truck cabs. Surfaces inoculated with organic material shown here are not reported in the current manuscript.

Surfaces were inoculated with either 1 mL of PRRSV (titer of 10^5^ TCID_50_/mL) or PEDV (titer of 10^5^ TCID_50_/mL). Surfaces were allowed to dry for 1 h prior to placement within an individual truck cab. When surfaces were ready for placement, surface coupons were placed within the truck cab with nitrile gloves changed in-between new surface coupons. Coupon placement was predetermined prior to the start of the study to ensure a consistent placement of surface coupons with each treatment and corresponded to the location within truck cabs from which the surfaces would naturally be located. Plastic coupon surfaces were placed on the dashboard, rubber surface coupons were placed on the floorboard, and fabric surface coupons were placed on the driver’s seat as shown in Figure 1. Surfaces shown include both surfaces with viral inoculum applied directly, or surfaces containing the presence of organic matter and viral inoculum. The results herein represent the results for clean surfaces only.

After surfaces were placed in the truck cab, a randomly assigned disinfectant treatment application was conducted. Independent disinfectant treatments were applied in each truck cab within each run, although each disinfectant treatment was performed in each truck cab for a total of 3 replicates for each disinfectant treatment. For the pump sprayer application, the applicator was positioned outside the truck cab on the driver’s side, and the liquid was applied in a snake-like application going from the front to the back of the cab, resulting in 50–90 g of disinfectant solution being used per treatment application. For the misting fogger application, the head of the fogger was angled and secured at 90° (parallel with the ground), placed in the passenger side seat, and aimed for the driver side of the truck cab. Once set in location, the flow rate was set to 2, turned on, the passenger door was closed, and the fogger was allowed to run for 5 min. The amount of disinfection used for this application ranged from 220 to 340 g. Once the application of the pump sprayer and the misting fogger was completed, wet application methods were allowed to dry for 15 min prior to environmental sample collection.

For the gaseous fumigation treatment, the plastic container was placed in the passenger’s seat, doors were closed, and fumigation was allowed to occur for 10 h after which the driver and passenger doors were opened, and the truck cab was allowed to dissipate for 1 h before environmental samples were collected. For the no chemical treatment and the 10 h downtime treatment, surface coupons were inoculated with virus, placed in truck cabs as previously mentioned, and they remained in the truck cab for approximately 15 min or 10 h, respectively, before sampling. Each combination of virus, surface, and disinfectant treatment was replicated three times.

### 2.4. Environment Sampling

Environmental samples were collected with cotton gauze as previously described [24]. Briefly, a 10 cm × 10 cm cotton gauze, pre-moistened with 5 mL PBS, was utilized to swab the surface coupon after treatment application. Environmental samples were transferred to the BSC, 20 mL of PBS was added to them, and they were inverted for 5–10 s and allowed to sit at room temperature for 1 h. The samples were then vortexed for 15 s, and the supernatant was transferred into 1.75 mL cryovials and 15 mL conical tubes. Samples were stored at −80 °C until PCR analysis. Once all samples were collected, surface coupons were discarded, and cabs were cleaned, sprayed with 1:256 glutaraldehyde, and allowed to dry for 20 min to prevent virus accumulation within the truck cabs.

### 2.5. Reverse Transcription Real-Time PCR Analysis

Reverse transcription real-time PCR (qRT-PCR) was conducted at the Molecular Research and Development Laboratory within the Kansas State Veterinary Diagnostic Laboratory. Fifty µL of supernatant from each sample was loaded into a deep-well plate and extracted using a Kingfisher Flex magnetic particle processor (Fisher Scientific, Pittsburg, PA, USA) and the MagMAX-96 Viral RNA Isolation kit (Life Technologies, Grand Island, NY, USA) according to manufacturer’s instructions with one modification, reducing the final elution volume to 60 μL. One negative extraction control consisting of all reagents except the sample (replaced with PBS buffer) was included in each extraction. Positive controls of each stock virus were also included with each extraction. Extracted RNA was frozen at −80 °C until assayed by qRT-PCR using a multiplex qRT-PCR assay developed by the KSU Molecular Research and Development Laboratory to simultaneously detect and quantify PRRSV and PEDV RNA. Analyzed values represent cycle threshold (Ct) at which virus was detected. If a sample had no detectable PRRSV or PEDV RNA, a sample was assigned a value of 45 as a total of 45 cycles were run for each sample. For result interpretation, greater Ct values indicate less detectable viral RNA.

### 2.6. Bioassay Analysis

A total of 16 treatments were selected for pig bioassay to assess the infectivity of virus present in them (Table 1). The bioassay procedure was conducted 13 months after surface inoculation and environmental sample collection. The project was approved through Iowa State University’s institutional animal care and use committee (Project #IACUC-22-082) and institutional biosafety committee (Project #22-045). A total of 48 crossbred, 10-day-old pigs of mixed sex were sourced from a single commercial, a crossbred farrow-to-wean herd with no prior exposure to PEDV and PRRSV. The Iowa State University Veterinary Diagnostic Laboratory confirmed all pigs to be negative for PRRSV using blood samples and PEDV, porcine deltacoronavirus, and transmissible gastroenteritis virus using fecal swabs via the respective real-time RT-PCR. Blood serum testing further confirmed that pigs were negative for PRRSV antibody using nucleocapsid protein-based ELISA and negative for PEDV antibody using an indirect fluorescent antibody assay. Pigs were allowed 3 days of acclimation prior to beginning of the bioassay. Three pigs were housed in a room, and each pig was challenged via an oral gavage of PEDV inoculum, an intramuscular (IM) injection, and an intranasal application of PRRSV. The oral gavage method was modeled similar to the described in the previous research [25], utilizing a 10 French feed tube and 60 mL syringe (10 mL/pig). For the PRRSV inoculum, 3 mL was administered to the muscle of the cervical region. For PRRSV intranasal inoculation, the tip of a 3 mL Luer slip syringe was inserted inside one of the pig’s nostrils, and 1 mL of inoculum was allowed to drip directly into the pig’s nostril. Rectal swabs were collected on days −3, 0, 4, and 7 post inoculation (dpi) from all pigs and tested for PEDV RNA using qRT-PCR. Serum samples were collected on −3, 0, 4, and 7 dpi from all pigs and tested for PRRSV RNA using qRT-PCR. Following humane euthanasia at 7 dpi, the small intestine, the cecum, an aliquot of cecal contents as described by Schumacher et al. [26], and lung samples were collected at necropsy. A negative bioassay was concluded if all rectal swabs, serum samples, lung tissue, and cecum contents had non-detectable levels of PEDV or PRRSV. If any samples had detectable RNA, the result would be considered a positive bioassay.

### 2.7. Statistical Analysis

Data were analyzed in a split-plot design with truck cab as the experimental unit for disinfectant treatment and surface coupons as the experimental unit for surface type (fabric, plastic, or rubber) and virus (PEDV or PRRSV). There were three replications per treatment. The Ct value of each sample was analyzed with ANOVA and *F*-test through the aov function in R programming language (R Foundation for Statistical Computing, Vienna, Austria). Fixed effects considered the disinfectant treatment, surface treatment, and virus type, while the random effect was truck cab defining it as the experimental unit for disinfectant treatment to account for the split-plot design. Results of Ct data are reported as least squares means ± standard error of the mean. All statistical models were evaluated using the visual assessment of studentized residuals, and assumptions appeared to be reasonably met. A Tukey multiple comparison adjustment was utilized to control Type I error rate. Results were considered significant at *p* ≤ 0.05 and marginally significant between *p* > 0.05 and *p* ≤ 0.10.

## 3. Results and Discussion

Both PEDV and PRRSV are considered endemic, or present at normal levels, within the swine-dense regions of the United States. Control of these and other pathogens are critical to maintain animal health and welfare. Given the significant economic impact of PRRSV and PEDV, continued efforts must be placed on the control of these pathogens. Both viruses share a similar structure being enveloped, positive-sense, single-stranded RNA viruses [9,10]. Importantly, because of their viral envelope, disinfection and inactivation is readily possible if correct procedures are implemented.

The transmission of PRRSV occurs through exposure via the respiratory and/or oral routes as well as through vertical transmission in gestating females [10], while the transmission of PEDV occurs through fecal–oral transmission [9]. Thus, for an effective control program, biosecurity efforts must be implemented to reduce the risk of these transmission events from occurring by focusing on controlling vectors including animal movement, supply entry into farms, personnel movement, and other fomites such as transportation vehicles.

The control of these viral pathogens requires a complex strategy addressing all possible routes of entry including animal movement, supply entry into farms, personnel movement, and other fomites such as transportation vehicles. Significant research efforts have focused on diagnostic testing to properly determine the health status of animals prior to movement, the decontamination of incoming supplies [27], the filtration of incoming air [28], and multiple investigations to address feed biosecurity and reduce the likelihood of pathogen introduction via incoming feed as reviewed by Stewart et al. [29].

It has been documented that transportation vehicles can contribute to the spread of both pathogens in addition to the direct contact routes of transmission [12,13,30,31]. Given this potential risk of viral transmission, there has been research documenting the benefits of power washing, disinfecting, and then heating livestock trailers to reduce the amount of potential infective material to limit the spread of disease to naïve pigs due to both PEDV and PRRSV [16,17,18,19,32]. However, most of this research has focused on how to reduce transmission in transportation with live animals and not on transportation vehicles that can frequently visit production sites without live animals, including feed delivery trucks. Furthermore, most research has focused on the inactivation of viruses on surfaces in an experimental setting, but little research has focused on the practical application of decontamination treatments in real-world settings using complex surfaces such as those found in the cab of semi-trucks that contain multiple surfaces as well as many surfaces with different orientations, making the practical application of disinfectants challenging.

There is evidence to suggest that when sampling for viruses or bacteria within a feed mill, a majority of the samples containing detectable pathogens were from the cab of the delivery truck, most likely due to the driver getting in and out of the cab for deliveries at production sites and potentially “tracking” virus back into the cab [11,14,15,22]. There are ways to mitigate this risk, including the use of shoe covers for each delivery or the application of disinfectant to the truck cab since the application of disinfectant has been successful for PEDV and PRRSV reduction in terms of livestock trailers; however, challenges exist when trying to transfer these procedures to truck cabs. The problems to consider when applying disinfectant to the truck cab are how to apply the disinfectant, the type of disinfectant (in terms of wet, dry, or fumigation), and the implications of disinfectant on the surfaces since there are various surface types present throughout the truck cab, unlike livestock trailers that are primarily made of metal. There is minimal research looking at different application methods and how these applications impact the amount of detectable virus within truck cabs on different surface types. Therefore, this study aimed to quantify the impact of commercially available disinfectants applied through different application methods on detectable viral RNA on fabric, plastic, and rubber surfaces inoculated with either PEDV or PRRSV and if detectable viral RNA could cause infection in susceptible pigs via swine bioassay.

There was no evidence of a disinfectant × surface × virus interaction (*p* = 0.959; Table 2), surface × virus interaction (*p* = 0.926), or disinfectant × virus interaction (*p* = 0.508).

There was a significant disinfectant × surface interaction (*p* < 0.0001; Table 3), indicating that the quantity of PEDV or PRRSV RNA detected on surfaces differed based on disinfectant treatment.

For rubber surfaces, environmental samples from the 10% bleach application had the least (*p* < 0.05) amount of PEDV or PRRSV RNA detected compared to the other disinfectant methods with Intervention via misting fumigation application as intermediate. For plastic and fabric surfaces, there was no evidence of a statistical difference between decontamination methods (*p* > 0.05). Furthermore, for the no disinfectant treatment, environmental swabs from fabric surfaces detected less PEDV or PRRSV RNA than that from plastic or rubber surfaces (*p* < 0.05). For the pump sprayer with Intervention disinfectant treatment, environmental swabs from fabric surfaces detected less (*p* < 0.05) PEDV or PRRSV RNA compared to that from rubber surfaces with plastic surfaces as intermediate. For the pump sprayer with 10% bleach disinfectant treatment, environmental swabs from fabric and rubber surfaces detected less PEDV or PRRSV RNA than that from plastic surfaces (*p* < 0.05). For the no chemical 10 h downtime disinfectant treatment, environmental swabs from fabric surfaces detected less PEDV or PRRSV RNA when compared to that from plastic or rubber surfaces (*p* < 0.05). Lastly, for the gaseous treatment 10 h downtime disinfectant application, environmental swabs from fabric surfaces detected less PEDV or PRRSV RNA when compared to that from plastic or rubber surfaces (*p* < 0.05). These results are similar to Muckey et al. [23], where there was also a treatment × surface interaction, indicating that when implementing disinfectant applications, the surface should be taken into consideration since surface material has the potential to influence the amount of detectable viral RNA. Given this commonality between research studies, there is some suggestion that surface type can influence the amount of detectable viral RNA recovered within sampling techniques.

The main effect of disinfection treatment was statistically significant (*p* = 0.016; Table 4), with environmental swabs collected after 10% bleach treatment, detecting less PEDV or PRRSV RNA (*p* < 0.05) when compared to environmental swabs from no disinfectant, Intervention via pump sprayers, and no chemical application 10 h downtime with all other disinfectant treatment applications being intermediate.

Disinfectants have been shown to reduce the amount of detectable PRRSV or PEDV RNA [16,17,18,33]. However, research suggests that the application of sodium hypochlorite, or bleach, results in the greatest reduction of PEDV, PRRSV, and other enteric viruses or bacteria, sometimes producing non-detectable test results for pathogens compared to other disinfectants or soaps used based on the surface material, which is consistent with the findings from this study [16,17,18,33,34,35].

The main effect of the surface was statistically significant (*p* < 0.0001), with environmental swabs from plastic surfaces detecting greater amounts of PEDV or PRRSV RNA when compared to those from rubber, and the samples from rubber detecting greater amounts of PEDV or PRRSV RNA when compared to those from fabric (*p* < 0.05). Rabuza et al. [36] reported that when sampling with cotton gauze, a methodology similar to that used in this experiment, the cotton gauze method extracted lower quantities of *Klebsiella pneumoniae* and *Staphylococcus aureus* from inoculated fabric surfaces when compared to other methods like contact plating, destructive elution methodology, or nondestructive elution methodology. For contact plating, the fabric sample had RODAC agar poured over it, so it could stick to the medium, while destructive or nondestructive elution methodologies are wash-off methods where microorganisms are eluted from the fabrics by shaking the fabrics for a specific time in an elution medium. Then, if the sample was assigned the nondestructive elution methodology, the sample was subjected to forced desorption by pressing the microorganism through the fabric without destroying the fabric. While contact plating, destructive elution methodology, or nondestructive elution methodology are employed to quantify bacteria on fabric surfaces, all methodologies require a fabric sample. These methodologies have their strengths, but there is minimal real-world application since pieces of a seat cover cannot be cut off from a seat within a truck cab. The advantage of the sampling methodology used in the current experiment is the ease of application in a practical field setting. Fabric surfaces have the potential to absorb and hold wet disinfectants for longer periods when compared to other non-absorptive surfaces like plastic or rubber, indicating that inherent properties of the surface could lend to longer disinfectant contact times. When consulting hospital procedures for disinfectant application, spraying disinfectants on various surfaces is a common solution for disinfection. However, excessive wetting of fabric surfaces is highly discouraged due to patient discomfort, and disinfectants will continuously leak out of fabric surfaces for extended periods [37]. It is uncertain which factor, either sampling method or the inherent properties of fabric, may have potentially impacted the Ct results from this study. These findings highlight the need for more research to be conducted on disinfectant application on fabric surfaces and applicable sampling methodology. The main effect of virus (*p* < 0.0001) was significant, with environmental swabs detecting greater amounts of PEDV RNA compared to the detection of PRRSV RNA (*p* < 0.05).

Supernatant from environmental samples of surfaces inoculated with PEDV and PRRSV after disinfectant treatment application were utilized in bioassay and failed to produce infectivity. To our knowledge, this is the first published study utilizing inoculum from environmental samples in which the positive control treatment groups (with Ct values ranging 26.4–37.2) did not cause clinical infection in pigs via bioassay. A potential explanation for the failure to produce infectivity is the time from sample collection and processing to the time of bioassay, potentially impacting infectivity factors for PEDV and PRRSV. For example, the length of time from sample collection to bioassay for this study was 13 months, while for the dust sample study, the length of time from sample collection to bioassay was 11 months [38]. More research is needed to understand the impacts of long-term storage on virus infectivity.

This study aimed to test disinfectant applications that could be implemented between feed deliveries or while feed trucks are loaded at the feed mill. Additionally, the data presented here provide value to a broader segment of the industry in comparing disinfectants through different applications that could be quickly implemented through a variety of truck cabs like those used in live-animal delivery, veterinary services, and other trucks associated with the swine and animal industries.

## 4. Conclusions

In conclusion, the disinfection of truck cabs has its challenges given the variety of surfaces present. The disinfectant treatments tested in this trial could only reduce the detectable amount of viral RNA and did not completely eliminate the viral genetic material across surface types. Based on this study and methodology utilized for bioassay, this amount of detectable viral RNA was not sufficient to cause infectivity in a swine bioassay. Additional research is warranted to refine methods of detecting infectious viruses across varying surfaces.

## Figures and Tables

**Table 1:** Summary of the 16 treatments chosen for live pig bioassay ^1^.

Surface Type	Disinfectant Treatment
Rubber (n = 8)	No disinfectant
	No chemical, 10 h downtime
	Gaseous fumigation, 10 h downtime
	Misting fumigation, Intervention
	Misting fumigation, Synergize
	Pump sprayer, Intervention
	Pump sprayer, Synergize
	Pump sprayer, 10% bleach
Plastic (n = 4)	No disinfectant
	No chemical, 10 h downtime
	Gaseous fumigation, 10 h downtime
	Pump sprayer, 10% bleach
Fabric (n = 4)	No disinfectant
	No chemical, 10 h downtime
	Gaseous fumigation, 10 h downtime
	Pump sprayer, 10% bleach

^1^ Supernatant from environmental samples taken after disinfectant application to a truck cab were used as the inoculum for the live pig bioassay. A total of 48 crossbred, 10-day-old pigs of mixed sex were administered 10 mL oral gavage of porcine epidemic diarrhea virus (PEDV) and 3 mL intramuscular (IM) injection, and 1 mL/nostril intranasal application of porcine reproductive and respiratory syndrome virus (PRRSV) after a three-day acclimation period.

**Table 2:** Effect of surface type, disinfectant, and virus on the detection of viral RNA during truck cab decontamination ^1^.

Item	Surface Type					
	Fabric		Plastic		Rubber	
	PEDV	PRRSV	PEDV	PRRSV	PEDV	PRRSV
Proportion PCR positive						
No disinfectant ^2^	3/3	3/3	3/3	3/3	3/3	3/3
Misting fumigation ^3^						
Intervention ^4^	3/3	3/3	3/3	3/3	2/3	2/3
Synergize ^5^	2/3	3/3	3/3	3/3	3/3	3/3
Pump sprayer ^6^						
Intervention	3/3	3/3	3/3	3/3	3/3	3/3
Synergize	2/3	3/3	2/3	3/3	3/3	3/3
10% Bleach ^7^	2/3	0/3	3/3	3/3	1/3	0/3
10 h downtime ^8^						
No chemical	3/3	2/3	3/3	3/3	3/3	3/3
Gaseous treatment ^9^	3/3	1/3	3/3	3/3	3/3	3/3
Cycle threshold ^10^						
No disinfectant	34.6	37.2	26.7	30.6	26.7	31.4
Misting fumigation						
Intervention	33.4	38.2	28.1	31.6	34.2	36.7
Synergize	36.2	36.4	29.7	34.2	30.3	33.3
Pump sprayer						
Intervention	34.8	37.5	28.3	31.3	28.8	32.0
Synergize	37.3	38.7	33.0	32.6	30.6	33.5
10% Bleach	40.7	45.0	26.7	31.2	41.2	45.0
10 h downtime						
No chemical	36.4	40.3	27.8	29.8	29.7	30.2
Gaseous treatment	36.8	44.4	28.3	31.9	28.6	33.2

^1^ Surfaces were inoculated with 1 mL of porcine epidemic diarrhea virus (PEDV) or porcine reproductive and respiratory syndrome virus (PRRSV), randomly placed within the truck cab, and subjected to a randomly assigned disinfectant treatment. Samples with no detectable RNA were assigned a value of 45. ^2^ Surfaces were inoculated with pure virus and allowed to sit within the truck cab for 15 min. These surfaces were not treated with a disinfectant application. ^3^ Truck cabs had a hurricane fumigation system placed in the passenger seat and directed toward the driver’s side. The hurricane fumigation system was filled with respective disinfectant and was allowed to run 5 min for each treatment. ^4^ 1:64 dilution of Intervention (Virox, Oakville, ON, USA). ^5^ 1:256 dilution of Synergize (Neogen Corp, Lexington, KY, USA). ^6^ Truck cabs had disinfectant applied with a conventional pump sprayer with the designated disinfectant. ^7^ Household bleach (10% dilution; The Clorox Company, Oakland, CA; 7.55% sodium hypochlorite). ^8^ Surfaces were inoculated with pure virus and allowed to sit within the truck cab for 10 h. These surfaces were not treated with a disinfectant application. ^9^ Truck cabs had gaseous chlorine dioxide (ProKure G; ProKure Solutions, Phoenix, AZ, USA) placed on their passenger side seats, and the truck cabs were allowed to fumigate for 10 h. ^10^ Disinfectant × surface × virus interaction, *p* = 0.959; SEM = 1.84.

**Table 3:** Effect of surface type and disinfectant on the detection of viral RNA during truck cab decontamination ^1^.

Item	Proportion PCR Positive			Ct Value ^2^		
	Fabric	Plastic	Rubber	Fabric	Plastic	Rubber
No disinfectant ^3^	6/6	6/6	6/6	35.9 ^c,d,e,f,g,h^	28.6 ^a,b^	29.0 ^a,b^
Misting fumigation ^4^						
Intervention ^5^	6/6	6/6	5/6	35.8 ^a,b,c,d,e,f,g,h^	29.8 ^a,b,c,d^	35.4 ^a,b,c,d,e,f,g,h^
Synergize ^6^	5/6	6/6	6/6	36.6 ^a,b,c,d,e,f,g,h^	31.9 ^a,b,c,d,e,f^	31.8 ^a,b,c,d,e,f^
Pump sprayer ^7^						
Intervention	6/6	6/6	6/6	36.1 ^b,d,e,f,g,h^	29.8 ^a,c^	30.4 ^a,b,c,d,e,f^
Synergize	5/6	5/6	6/6	38.0 ^e,f,g,h^	32.8 ^a,b,c,d,e,f,g^	32.0 ^a,b,c,d,e,f^
10% Bleach ^8^	2/6	6/6	1/6	42.9 ^h^	29.0 ^a,b,c,d^	43.1 ^h^
10 h downtime ^9^						
No chemical	5/6	6/6	6/6	38.4 ^f,g,h^	28.8 ^a,b,c,d^	30.0 ^a,b,c,d^
Gaseous treatment ^10^	4/6	6/6	6/6	40.6 ^g,h^	30.1 ^a,b,c,d,e^	30.9 ^a,b,c,d,e,f^

^1^ Surfaces were inoculated with 1 mL of porcine epidemic diarrhea virus (PEDV) or porcine reproductive and respiratory syndrome virus (PRRSV), randomly placed within the truck cab, and subjected to a randomly assigned disinfectant treatment. Samples with no detectable RNA were assigned a value of 45. ^2^ Disinfectant × surface, *p* < 0.0001; SEM = 1.45. ^3^ Surfaces were inoculated with pure virus and allowed to sit within the truck cab for 15 min. These surfaces were not treated with a disinfectant application. ^4^ Truck cabs had a hurricane fumigation system placed in the passenger seat and directed toward the driver’s side. The hurricane fumigation system was filled with respective disinfectant and was allowed to run 5 min for each treatment. ^5^ 1:64 dilution of Intervention (Virox, Oakville, ON, USA). ^6^ 1:256 dilution of Synergize (Neogen Corp, Lexington, KY, USA). ^7^ Truck cabs had disinfectant applied with a conventional pump sprayer with the designated disinfectant. ^8^ Household bleach (10% dilution; The Clorox Company, Oakland, CA, USA; 7.55% sodium hypochlorite). ^9^ Surfaces were inoculated with pure virus and allowed to sit within the truck cab for 10 h. These surfaces were not treated with a disinfectant application. ^10^ Truck cabs had gaseous chlorine dioxide (ProKure G; ProKure Solutions, Phoenix, AZ, USA) placed on their passenger side seat, and the truck cabs were allowed to fumigate for 10 h. ^a–h^ Means lacking common superscripts differ, *p* < 0.05.

**Table 4:** Main effects of disinfectant, surface type, and virus on the detection of viral RNA during truck cab decontamination ^1^.

Item	Proportion PCR Positive	Ct Value	SEM	*p*=
Disinfectant			1.11	0.016
No disinfectant ^2^	18/18	31.2 ^a^		
Hurricane fumigation ^3^				
Intervention ^4^	16/18	33.7 ^a,b^		
Synergize ^5^	17/18	33.4 ^a,b^		
Pump sprayer ^6^				
Intervention	18/18	32.1 ^a^		
Synergize	16/18	34.3 ^a,b^		
10% Bleach ^7^	9/18	38.3 ^b^		
10 h Downtime				
No chemical ^8^	17/18	32.4 ^a^		
Gaseous treatment ^9^	16/18	33.9 ^a,b^		
Surface Type			0.53	<0.0001
Fabric	39/48	38.0 ^c^		
Plastic	47/48	30.1 ^a^		
Rubber	41/48	32.8 ^b^		
Virus			0.48	<0.0001
PEDV	62/72	32.0 ^a^		
PRRSV	62/72	35.3 ^b^		

^1^ Surfaces were inoculated with 1 mL of porcine epidemic diarrhea virus (PEDV) or porcine reproductive and respiratory syndrome virus (PRRSV), randomly placed within the truck cab, and subjected to a randomly assigned disinfectant treatment. Samples with no detectable RNA were assigned a value of 45. ^2^ Surfaces were inoculated with pure virus and allowed to sit within the truck cab for 15 min. These surfaces were not treated with a disinfectant application. ^3^ Truck cabs had a hurricane fumigation system placed in the passenger seat and directed toward the driver’s side. The hurricane fumigation system was filled with respective disinfectant and was allowed to run 5 min for each treatment. ^4^ 1:64 dilution of Intervention (Virox, Oakville, ON, USA). ^5^ 1:256 dilution of Synergize (Neogen Corp, Lexington, KY, USA). ^6^ Truck cabs had disinfectant applied with a conventional pump sprayer with the designated disinfectant. ^7^ Household bleach (10% dilution; The Clorox Company, Oakland, CA, USA; 7.55% sodium hypochlorite). ^8^ Surfaces were inoculated with pure virus and allowed to sit within the truck cab for 10 h. These surfaces were not treated with a disinfectant application. ^9^ Truck cabs had gaseous chlorine dioxide (ProKure G; ProKure Solutions, Phoenix, AZ, USA) placed on their passenger side seat, and the truck cabs were allowed to fumigate for 10 h. ^a,b,c^ Means within main effect lacking common superscripts differ, *p* < 0.05.

## Data Availability

All data generated or analyzed during this study are included in this published article.
